# Improving knowledge, attitudes and beliefs: a cross-sectional study of postpartum depression awareness among social support networks during COVID-19 pandemic in Malaysia

**DOI:** 10.1186/s12905-022-01795-x

**Published:** 2022-06-11

**Authors:** Rania Nafi’ Suleiman Alsabi, Alif Firdaus Zaimi, Thanusha Sivalingam, Nurul Nazirah Ishak, Aishah Siddiqah Alimuddin, Rima Aggrena Dasrilsyah, Nurul Iftida Basri, Amilia Afzan Mohd Jamil

**Affiliations:** 1 Department of Nursing, Faculty of Medicine and Health Sciences, University Putra Malaysia, 43400 Serdang, Selangor Darul Ehsan Malaysia; 2 Faculty of Medicine and Health Sciences, Universiti Putra Malaysia Serdang, 43400 Serdang, Malaysia; 3 Department of Psychiatry, Hospital Pengajar Universiti Putra Malaysia, 43400 Serdang, Selangor Darul Ehsan Malaysia; 4 Department of Psychiatry, Faculty of Medicine and Health Sciences, University Putra Malaysia, 43400 Serdang, Selangor Darul Ehsan Malaysia; 5 Department of Obstetrics and Gynaecology, Faculty of Medicine and Health Sciences, University Putra Malaysia, 43400 Serdang, Selangor Darul Ehsan Malaysia; 6 Department of Obstetrics and Gynaecology, Hospital Pengajar Universiti Putra Malaysia, 43400 Serdang, Selangor Darul Ehsan Malaysia

**Keywords:** Postpartum depression, Knowledge, Attitudes, Beliefs, Social support networks

## Abstract

**Background:**

Postpartum depression (PPD) is the most prevalent mental health disorder after childbirth, notably during the COVID-19 pandemic. In addition, PPD is known to have a long-term influence on the mother and the newborn, and the role of social support network is crucial in early illness recognition. This study aims to evaluate the social support networks’ level of knowledge, attitudes and beliefs regarding PPD and examine their sociodemographic variables and exposure to the public information relating to PPD during the COVID-19 pandemic in Malaysia.

**Methods:**

A cross-sectional study was conducted via an online Google Form disseminated to people in Klang Valley through WhatsApp, Email, Facebook, Instagram and other available social media among postpartum women’s social support networks aged 18 years and living in the Klang Valley area (*N* = 394). Data were collected from 1 March to 5 July 2021 and analysed using the Mann–Whitney *U*-test and generalised linear mixed models.

**Results:**

During the COVID-19 epidemic in Klang Valley, most participants had good knowledge, negative attitudes and awareness of PPD. Marital status, gender and parity all had significant correlations with the amount of awareness regarding PPD. Ethnicity, gender, parity and educational level showed significant association with attitude towards PPD. No significant relationship was noted between sociodemographic variables and PPD beliefs. Public awareness of PPD was also associated with knowledge and attitude towards it.

**Conclusions:**

A significant positive knowledge, negative attitude and negative awareness level of PPD exist among social support networks for postnatal women. However, no significant effect of belief on PPD awareness level was noted.

**Implications:**

Insight campaigns and public education about PPD should be conducted to enhance postnatal mothers’ awareness and knowledge. Postnatal care, mental check-ups and counselling sessions for the new mothers are recommended. In future studies, a closer assessment of postpartum social support, variances and similarities across women from diverse racial/ethnic origins is critical.

**Strengths and limitations:**

This cross-sectional study is one of the early studies on the area of PPD in the Malaysian region during COVID-19. Numerous data have been collected using low-cost approaches using self-administered surveys through Google Forms in this research.

## Background

Postpartum depression (PPD) is the most common predisposing illness for women in their reproductive years [1]. As a result of studies and clinical practices, PPD was defined as the incidence of despair immediately following childbirth [2]. However, the severity ranged from moderate to psychotic mental manifestations and illnesses throughout pregnancy and the postoperative period [3–5]. As a result, disorders (e.g. PPD, family, social, economic and occupational collapses) can occur [6]. PPD could have adverse effects on the mother’s mental and physical health, her marital closeness and connection with the newborn and infant growth [7]. Mothers with PPD are less likely to welcome, engage and communicate with their newborns [4, 8–10] and are less in harmony with their infants [4, 11, 12].

Although no specific PPD classifications exist in the Diagnostic and Statistical Manual, the American Psychiatric Association [13] did register a word for when PPD occurs within 4 weeks after delivery, called *postpartum onset* [14]. In addition to moodiness, sadness, amnesia, irritability and uneasiness [4, 15], PPD has been linked to diarrheal disease, incomplete immunisation and impaired cognitive development in young children [16–18]. According to a rough estimate, only 20% of women experiencing PPD symptoms disclose those manifestations to health experts [19].

PPD is common in the first 3 months after childbirth, according to a global systematic analysis of 59 studies [7]. In Malaysia, the PPD prevalence varies from 14.3 to 31.7% [20]. One study found that PPD frequency in a rural region of Northern Peninsular Malaysia was 9.4% [6, 21]. In reviews and epidemiological studies encompassing a range of cultures worldwide [4, 22], an increase in PPD incidence has been reported [16]. Asians, Europeans and North Americans have different ways of expressing depression, e.g. physical and affective symptoms [4, 23].

The perinatal mental healthcare sector must be improved to avoid serious PPD consequences from happening, e.g. self-suicide and filicide (the act of killing children) [19]. Women with PPD need a caring spouse to help them through this challenging period [24].

However, the social support intervention did not appear to treat PPD in adolescents at 6 weeks postpartum but play important role in early detection as well as continuous support for PPD sufferers [25]. PPD was more likely to occur if social support was not provided during pregnancy [26]. Women’s mental health was greatly affected by social support networks, e.g. partner and family [27].

For many years, researchers have attempted to define and quantify the idea of social support. For the sake of this definition, social support may be broadly defined as a selfless act of kindness performed by one person (the donor) for the benefit of another (the receiver) and marked by the recipient’s immediate or delayed positive emotional reaction [28]. Family members, friends, husband/wife and other people can all give a voluntary act. It can be informational, physical, emotional (e.g. empathy and caring), instrumental and appraisal [25].

In the 1970s, pioneering studies showed that the social environment had a direct effect on health outcomes to the extent that social support may prevent illness [29]. Individuals who have more social support are more likely than those with less social support to live longer, according to a wide range of recent studies [30]. For childcare and domestic assistance in the United Kingdom, family members, e.g. maternal grandmothers, have been highlighted as key players [31]. In the USA, this assistance may come from a range of people, including one’s spouse, family, friends and healthcare professionals [32]. Family support networks have played an important role in helping families cope with the immediate needs brought on by the COVID-19 pandemic, e.g. financial resources and educational support for many vulnerable families who are likely to be most affected by the pandemic’s long-term economic, educational, health and well-being consequences [33, 34].

The COVID-19 pandemic had direct psychological and social consequences on postpartum mothers. In Malaysia, the Movement Control Order (MCO) [35] and social distancing guidelines as a result of COVID-19 imposed unusual limits on social connections in the perinatal population, the date of which corresponded with a period when these people may depend significantly on social support networks [32, 33]. Women who were pregnant or had just given birth were more likely to limit the size of their social groups during COVID-19 [34]. Many women were deprived of postpartum festivities and get-togethers because of social distancing norms that prohibited them from participating in such activities [35].

In Malaysia, reports exist on the COVID-19 pandemic’s effects on mental health, including sadness and anxiety. Stressors, e.g. the COVID-19 pandemic, exacerbate the adaptation and transition to motherhood, which is already difficult for women. PPD has been previously linked to a small number of biological, psychological, social and cultural variables [42], and a strong indication exists that numerous studies throughout the lockdown during the COVID-19 pandemic focused on the general population [36, 37], economics [38, 39], environment [40, 41]. However, no thorough study was done on PPD knowledge, attitudes and beliefs.

The PPD prevalence in the state of Kelantan was 22.8% and 20.7% at 1 and 4–6 weeks postpartum, respectively, and is poorly understood by the general public [21, 43–46]. Previous studies showed that most participants could not accurately identify PPD prevalence and a significant symptom (severe sorrow and irritability for more than 15 days), a diagnostic criterion for PPD following DSM-5 [13, 43]. This issue will likely become even more complicated if Malaysian adults ≥ 18 years old providing social support to postpartum mothers in Klang Valley are unaware of PPD. Lack of information about PPD may lead to feelings of uncertainty, loneliness and difficulty expressing emotional challenges, which will prevent women from seeking professional treatment to deal with the emotional issues that are incredibly prevalent in PPD. Thus, PPD symptoms will worsen over time and result in mortality [43, 47, 48].

During the COVID-19 outbreak in the Klang Valley, evaluating the degree of awareness about PPD among social support networks is crucial. This study aims to assess social support networks’ knowledge, attitudes and beliefs about PPD during the COVID-19 epidemic and examine the relationship between exposure to the public information about PPD and knowledge, attitude and belief about PPD in the Klang Valley, Malaysia.

## Methods

### Study design

Ethical approval was obtained from the Ethics Committee for Research Involving Human Subject (JKEUPM) with reference number JKEUPM-2021-096. The possible level of knowledge, attitudes and beliefs about PPD for social support networks of postpartum women was investigated as part of a large-scale study in the Klang Valley area of Malaysia during the COVID-19 pandemic.

The cross-sectional descriptive study used a web-based questionnaire. The questionnaire was sent to respondents using online Google Forms and promoted on various social media platforms like Facebook, Instagram, Twitter, WhatsApp and Messenger. The questionnaire was directed at Malaysians,more of equal to 18 years old, who may function as social support networks (husbands, friends, parents and in-laws as well as siblings) for postpartum women during the COVID-19 epidemic. These inclusion criteria were mentioned in the information sheet, and the questionnaires include checking questions to ensure that the participants are among the social networks for postpartum women (i.e. husbands, friends, parents and in-laws as well as siblings).

Pregnant and postpartum women were excluded from this study. This study used a convenience sampling strategy. Recruitment for this study was based on participants who satisfied all inclusion criteria and consented to take part in the survey. On social media networks (e.g. Facebook and Instagram as well as WhatsApp and Messenger), the questionnaire was turned into a Google Form and sent out.

Participants’ consent for participation in the study could be secured by presenting a brief explanation of the study on the first questionnaire page. A valid email address was required when participants accepted to participate in the survey. Automatically, the replies were stored in Google Form and used for future data collection, and the survey was limited to respondents who were at least 18 years old and lived in the Klang Valley. Online access to the questionnaire was accessible from March 1 to 5 July 2021.

The research questions of this study were:

- What is the level of knowledge, attitude and belief of PPD among social support networks (family, relatives and friends) in the Klang Valley during the COVID-19 pandemic?
- Is there any association between exposure to public awareness of PPD and knowledge, attitude and belief on PPD among social support networks in Klang Valley during the COVID-19 pandemic?

### Questionnaire

The questionnaire is divided into two sections. The first section contained 11 items that gathered information on the respondents’ sociodemographic and socioeconomic characteristics, including their age group, gender, ethnic origin, home location, marital status, parity, age of their last child, monthly income, educational level, professional status and employment status as a healthcare worker. Each question had a list of possible answers, and respondents were required to select the most acceptable ones.

The second component had a total of four major parts built up.

#### Part A (knowledge assessment questionnaire)

These items were adapted from a study conducted among the Portuguese population by Branquinho et al. [43] and assessed PPD knowledge and included 16 items that assessed prevalence, symptoms, risk factors, consequences and treatments for PPD. Three options were given for each question (true, false or not sure). A one-point penalty exists for each inaccurate or *not sure* response. The lowest possible score was 0, and the highest possible cumulative score was 16. The median total knowledge score was determined by adding the scores in each domain. The median total knowledge score was 8. The findings were then stated as being above or below the median score. A score of ≤ 8 and 8–16 shows a poor and strong knowledge of PPD, respectively.

#### Part B (attitudes assessment questionnaire)

These items were adapted from the previous study [49] and collected data on attitudes toward PPD and contained 17 items designed to measure an individual’s views toward PPD. Five possible answers to each item (strongly disagree, disagree, neutral, agree and strongly agree). Answers were then split into three categories throughout the scoring process: disagree (strongly disagree and disagree), neutral and agree (agree and strongly agree). A total of 0 points were awarded for *disagree*, 1 point for *neutral* and 2 points for *agree* for items 1–16. Zero points for *agree* and two points for *disagree were noted* for item 17. The maximum score that any respondent may achieve is 34. The median attitude score was calculated by adding the scores for each category, which is 17. The results were then expressed as above or below the median score: a score less than the median (17) suggests a favourable attitude toward PPD, whereas a score more than the median (17) shows a negative attitude toward PPD.

#### Part C (belief assessment questionnaire)

This included 10 items that were adapted from a study conducted among nurses in maternal and child health clinics assessing knowledge, beliefs and practices regarding the PPD screening and treatment by Kang et al. [49]. This particular section measures the respondents’ belief in PPD. The items were rated on a five-point Likert scale (strongly disagree, disagree, neutral, agree and strong agree). The five-point Likert scale was then recategorised into three groups: strongly disagree and disagree were merged to form the *disagree* group, *strongly agree* and *agree* were combined to form the *agree* group and *neutral* was retained as the *neutral* category. All 10 items differed depending on the number of beliefs toward PPD, which may be either negative or positive. Each positive belief response, each neutral answer and each negative belief received 2, 1 and 0 points, respectively. The lowest and highest possible scores were 0 and 20, respectively. By summarising all answers in the belief category, the median belief score was 10. Consequently, the findings were categorised as either above or below the median score, and all scores below the median score (10) suggest a negative degree of PPD belief.

#### Public education campaigns assessment questionnaire

This is collected as part of the questionnaire’s secondary component. Two items in the components assess the exposure of the participants to any information or campaign about postpartum depression during the COVID-19 pandemic and the perception of the participants on the usefulness of this campaign as part of the PPD knowledge transfer. Permission for translation was obtained from the author of the original questionnaire manuscript. The original English version of the questionnaire was translated into Malay by two Malay–English translators who were unfamiliar with the questionnaire. Controversial Malay wordings were discussed with the experts in the field (obstetricians and psychiatrists), and revisions were made.

### Data collection

The data was gathered using questionnaires created with Google Forms and sent to Klang Valley residents via WhatsApp, Email, Facebook, Instagram and other available social media platforms. Two languages were used in the questions (Malay and English) and were conducted online to complete the form at their convenience. Consent was then obtained by presenting the study’s brief introduction on the first questionnaire page (Google Form).

A valid email address was required when participants accepted to participate in the survey. Automatically, their replies were stored in Google Form and used for future data collection. No information about the participants will be disclosed to other parties. The data collected from Google Form answers were automatically categorised and it reduced the chance of making an error when analysing data.

### Sample size estimation

To estimate sample size of a proportion of one group:

$$n = \frac{{z_{1 - \alpha /2}^{2} P \left( {1 - P} \right)}}{{d^{2} }}$$

n = sample size, Z= z value (e.g., value 1.96 for 95% confidence level), Z^1-α/2^ = 95% of confidence interval at 1.96 [49]. P = Proportion of family members (social support network) who have shown a good level of knowledge/positive attitude towards women with PPD [50]. D = Precision (5% margin of error) [49].when P is proportion of family members (social support network) who have shown a positive attitude towards women with PPD = 69.7%

$$\begin{aligned} n = &\, \frac{{\left( {1.96} \right)^{2} \left( {0.697} \right)\left( {1 - 0.697} \right)}}{{\left( {0.05} \right)^{2} }} \\ = &\, {324}.{5} \approx { }325 \\ = &\, {324}.{5} \\ \end{aligned}$$

By considering risk of dropout of 20%,

$$\begin{aligned} {\text{n}} = &\, {325 } + \, \left( {{2}0\% {\text{ of 325}}} \right) \\ = &\, {325 } + \, \left( { \approx {65}} \right) \\ = & \,{\mathbf{390}}\,{\text{participants}}. \\ \end{aligned}$$

### Validity and reliability

The validity of the questionnaire’s content was established by modifying questions from prior surveys to control the quality of the questionnaire. First, questions in Sects. “Background” and “Methods” (parts A, B and D) were altered from the original study performed among the general Portuguese population [43]. Questions in Sect. “Methods”(part C) were adapted from a study conducted among nurses in maternal and child health clinics assessing knowledge, beliefs and practices related to PPD [49]. The questionnaire was used with the authors’ permission.

Second, pre-testing of the surveys was conducted online by sending the questionnaire to 10% of the sample size or about 50 participants over WhatsApp, requesting that they complete the questionnaire and provide comments on any issues they had while completing the questionnaire. The population for the pre-test is similar to the study population and they are not allowed to participate in the actual study.

Third, Cronbach’s alpha was calculated for PPD knowledge (0.772), attitudes (0.768) and beliefs (0.711) to confirm that the instrument’s internal consistency was > 0.7. The Cronbach’s alpha coefficient was also adapted from a previously completed study for various portions of the questionnaire. Section “Methods” (part A) had a Cronbach’s alpha coefficient of 0.72, and Section “Methods” (part B) had a Cronbach’s alpha of 0.77 [43].

Fourth, the content validation of the questionnaire was done through experts meeting participated by experts in postpartum care and management of postpartum depressive women.

### Data analysis

The Statistical Package for the Social Sciences (SPSS 26.0) was used to evaluate the data. The normality test could not be done because independent variables were not continuous data. Descriptive analysis was used to determine the frequency and percentage of respondents for each component (sociodemographic variables, knowledge, attitudes and beliefs about PPD).

The mean and standard deviation were computed to explore if the data were normally distributed, and the median and interquartile range if it was non-normally distributed. The Mann–Whitney *U*-test was used to examine the relationship between sociodemographic variables (categorical data) and knowledge, attitudes and beliefs about PPD (continuous data) among social support networks in Klang Valley.

For the next step, a generalised linear mixed model (GLMM) was undertaken to evaluate the variables of knowledge, attitudes and beliefs about PPD among social support networks during the COVID-19 pandemic in Klang Valley. GLMMs is a statistical model that extends the standard general linear model to incorporate non-normally distributed variables, non-linear relationships and data with dependencies. Continuous medical journals are used for modelling quantitative data or counts in recent years. According to the current guidelines, the quality of reporting may be improved in terms of the outcomes of the analysis, methods of estimation, validation and model selection [51].

## Results

The Google Form questionnaire that was distributed using online Google Forms and promoted on various social media platforms like Facebook, Instagram, Twitter, WhatsApp and Messenger, was answered by 445 participants. However, only 394 were included in the study because the other respondents were not from Klang Valley.

### Sociodemographic characteristics of respondents

Table 1 shows the sociodemographic characteristics distribution of the respondents in this study among social support networks during the COVID-19 pandemic in Klang Valley, Malaysia. Of the 394 respondents who completed the questionnaire, 73.4% were female and 90.9% belonged to the 18–39 year-old group. Regarding the racial background of the respondents, the majority were Malay respondents (75.1%), single (85.5%), have no children (86.5%) and had advanced education when completing the survey (89.1%). Unexpectedly, 74.1% of respondents reported non-exposure to PPD knowledge previously.

**Table 1:** Distribution of sociodemographic factors of respondents

Variables	Frequency	Percentage (%)
*Age group*		
18–39	358	90.9
> 40	36	9.1
*Gender*		
Male	105	26.6
Female	289	73.4
*Ethnicity*		
Malay	296	75.1
Non-Malay (Chinese, Indian, Others)	98	24.9
*Marital status*		
Single	337	85.5
Married	57	14.5
*Parity*		
Yes	53	13.5
No	341	86.5
*Educational level*		
Early (None, Primary and Secondary)	43	10.9
Advanced (Tertiary)	351	89.1
*Exposure to public knowledge on PPD*		
Yes	102	25.9
No	292	74.1

### Knowledge, attitude and beliefs on postpartum depression awareness

#### Frequencies of knowledge on postpartum depression awareness

According to the survey, as shown in Table 2, the majority of respondents (90.1%, *n* = 355) correctly answered how to overcome PPD during the COVID-19 pandemic, 81% (*n* = 319) answered that hormonal changes can cause PPD and 86% (*n* = 339) accurately identified professional therapy during the COVID-19 pandemic as necessary. Because of the non-normal distribution of knowledge scores among the respondents, the median [interquartile range (IQR)] of the data was set at 10.00 (4.00), and 76.4% (*n* = 301) of the respondents scored above the median total knowledge score (8/16), indicating strong PPD knowledge.

**Table 2:** Distribution of the respondents regarding knowledge on PPD among social support networks during COVID-19 pandemic in Klang Valley, Malaysia

No	Items	Correctn (%)	Incorrect n (%)
1	The occurrence of postpartum depression is higher during COVID-19 pandemic	212 (53.8)	182 (46.2)
2	Severe dejection and irritability, more than 15 days postpartum, are manifestations of postpartum depression	256 (65.0)	138 (35.0)
3	To overcome postpartum depression, the motivation of family and friends is a must during COVID-19 pandemic	355 (90.1)	39 (9.9)
4	Hormonal changes can cause postpartum depression	319 (81.0)	75 (19.0)
5	Women with postpartum depression only think about self-harm	133 (33.8)	261 (66.2)
6	Psychological intervention is productive in treating postpartum depression during COVID-19 pandemic	286 (72.6)	108 (27.4)
7	Women with symptoms of depression and anxiety during pregnancy tend to have postpartum depression	241 (61.2)	153 (38.8)
8	Women with postpartum depression cannot respond well to the infant's needs	242 (61.4)	152 (38.6)
9	Professional help is a need in treating postpartum depression during COVID-19 pandemic	339 (86.0)	55 (14.0)
10	The possibility of harming infants is high for women with postpartum depression	252 (64.0)	142 (36.0)
11	Women with postpartum depression usually have loss of appetite	207 (52.5)	187 (47.5)
12	Sleep disturbance occurred in women with postpartum depression	278 (70.6)	116 (29.4)
13	The women with postpartum depression do not communicate well with their parents and children	242 (61.4)	152 (38.6)
14	Women with postpartum depression cannot be diagnosed by general practitioner	62 (15.7)	332 (84.3)
15	Only women with a history of psychological problems or who did not wish to become pregnant can develop postpartum depression	207 (52.5)	187 (47.5)
16	Supplement and vitamin are recommended in treating postpartum depression	56 (14.2)	338 (85.8)

Frequency (n, %) of respondents answered correctly are presented in black

However, 66.2% (*n* = 261) of the respondents answered wrongly that women with PPD only think about self-harming when they are depressed. Moreover, up to 84.3% of respondents replied erroneously, stating that their family doctor cannot diagnose women with PPD. Unexpectedly, 85.8% (*n* = 338) did not realise that supplements and vitamins are recommended for treating PPD.

#### Frequencies of attitudes on postpartum depression awareness

The attitude score was not normally distributed with an asymmetrical curve among the respondents, and the median (IQR) was 14.00 (7.00). The median score for attitude was 17, while the maximum score was 34. The majority of the 394 (*n* = 261, 66.2%) respondents had a positive attitude about PPD. During the COVID-19 epidemic in Klang Valley, Malaysia, 33.8% (*n* = 133) of the respondents had a negative attitude towards PPD.

Table 3 displays the participants’ degree of agreement with 17 items assessing the attitude of the respondents on PPD among social support networks during the COVID-19 pandemic and showed that 84.3% (*n* = 332), 70.3% (*n* = 227) and 72.1% (*n* = 284) of the respondents thought that women with PPD do not choose to get PPD, believed that all postpartum women should undergo PPD screening and agreed that PPD is not a sign of weakness, respectively*.* Moreover, 78.4% (*n* = 309), 73.1% (*n* = 288) and 72.3% (*n* = 285)of the respondents disagreed that PPD is not a serious problem, believed that PPD did not exist in previous generations and reported that it is better if no one knows a woman is suffering from PPD, respectively.

**Table 3:** Distribution of the respondents regarding attitude on PPD among social support networks during the COVID-19 pandemic in Klang Valley, Malaysia

No	Items	Disagree (1–2) n (%)	Neutral (3) n (%)	Agree (4–5) n (%)
1	Postpartum depression is common	72 (18.3)	147 (37.3)	175 (44.4)
2	Women with postpartum depression cannot be good mothers	220 (55.8)	87 (22.1)	87 (22.1)
3	Postpartum depression is not a serious problem	309 (78.4)	44 (11.2)	41 (10.4)
4	Postpartum depression is a common fatigue and difficulty after childbirth	116 (29.4)	145 (36.8)	133 (33.8)
5	Women know, by nature, how to look after a baby	139 (35.3)	101 (25.6)	154 (39.1)
6	Women with postpartum depression hate their babies	157 (39.8)	130 (33.0)	107 (27.2)
7	Women have postpartum depression because they have unrealistic expectations about caring for a baby	98 (24.9)	159 (40.4)	137 (34.8)
8	Postpartum depression does not exist in previous generations	288 (73.1)	73 (18.5)	33 (8.4)
9	Postpartum depression is not a sign of weakness	26 (6.6)	84 (21.3)	284 (72.1)
10	Women suffer postpartum depression because they are not ready to make the sacrifice needed while caring for a child	150 (38.1)	139 (35.3)	105 (26.6)
11	Postnatal depression will go away on its own as the baby grows	153 (38.8)	169 (42.9)	72 (18.3)
12	There is no justification to get postpartum depression when women decide to give birth	145 (36.8)	136 (34.5)	113 (28.7)
13	Women do not choose to get postnatal depression	14 (3.6)	48 (12.2)	332 (84.3)
14	Although women experience postpartum depression, they must endure it without medical help	234 (59.4)	78 (19.8)	82 (20.8)
15	Women suffer postpartum depression because they are not ready to be a mother	164 (41.6)	137 (34.8)	93 (23.6)
16	It is better if no one knows a woman is suffering from postpartum depression	285 (72.3)	59 (15.0)	50 (12.7)
17	All postpartum women should undergo postpartum depression screening	31 (7.9)	86 (21.8)	277 (70.3)

#### Frequencies of beliefs on postpartum depression awareness

The belief score was not normally distributed among the respondents. Therefore, the median (IQR) was 12.00 (4.00). The median overall knowledge score was 10 out of a possible 20 points, and 84.8% (*n* = 334) of the participants scored above this number, indicating a positive belief about PPD. Based on the survey results, as shown in Table 4, most of the respondents (77.9%, *n* = 307) agreed on the necessity of regular postnatal mental health follow-ups during pandemic periods, while as many as 78.4% (*n* = 3 09) of the respondents believed that PPD is not genuine but merely a myth.

**Table 4:** Distribution of the respondents regarding belief on PPD among social support networks during the COVID-19 pandemic in Klang Valley, Malaysia

No	Items	Frequency (n = 394)	Percentage (%)
1	*Postpartum depression incidence increased during the COVID-19 pandemic*		
	Disagree (1–2)	8	2.0
	Neutral (3)	126	32.0
	Agree (4–5)	260	66.0
2	*Postpartum depression is not natural but just a myth*		
	Disagree (1–2)	309	78.4
	Neutral (3)	52	13.2
	Agree (4–5)	33	8.4
3	*The practice of mothers not talking about depression to doctors in our culture*		
	Disagree (1–2)	61	15.5
	Neutral (3)	125	31.7
	Agree (4–5)	208	52.8
4	*Postpartum depression is not fatal*		
	Disagree (1–2)	240	60.9
	Neutral (3)	101	25.6
	Agree (4–5)	53	13.5
5	*Mothers with postpartum depression prefer to seek alternative treatment for their depression*		
	Disagree (1–2)	34	8.6
	Neutral (3)	186	47.2
	Agree (4–5)	174	44.2
6	*Postpartum depression screening should be done online during the COVID-19 pandemic*		
	Disagree (1–2)	51	12.9
	Neutral (3)	129	32.7
	Agree (4–5)	214	54.3
7	*Screening for postpartum depression takes too much time*		
	Disagree (1–2)	88	22.3
	Neutral (3)	222	56.3
	Agree (4–5)	84	21.3
8	*Screening for postpartum depression is costly*		
	Disagree (1–2)Neutral (3)	76,234	19.359.4
	Agree (4–5)	84	21.3
9	*Regular postnatal mental health follow-ups in clinics are necessary even during pandemic periods*		
	Disagree (1–2)	10	2.5
	Neutral (3)	77	19.5
	Agree (4–5)	307	77.9
10	*It is difficult to get proper medical aid for postpartum depression during the COVID-19 pandemic*		
	Disagree (1–2)	55	14.0
	Neutral (3)	147	37.3
	Agree (4–5)	192	48.7

### Inferential statistics

#### Sociodemographic factors, postpartum depression awareness and knowledge

A relationship between sociodemographic variables and exposure to PPD with knowledge scores among social support networks during the COVID-19 pandemic in Klang Valley, Malaysia, using the Mann–Whitney *U*-test, was noted in Table 5 (*p* value < 0.05). A significant difference was noted between PPD knowledge scores (*U* = 7,836, unmarried = 75, single = 337, *p* = 0.025) for the married group compared to the single group. The median overall knowledge score was 11 of a possible 20 points for the married group compared to 10 for the single group suggesting that the marital status was significant.

**Table 5:** Association between sociodemographic factors and exposure to PPD with knowledge score by using Mann–Whitney U test (n = 394)

Variables	Freq., n (%)	Median (IQR)	Mann–Whitney U	p-value
*Marital status*				
Single	337 (85.5)	10.00 (4.00)	7836.000	0.025*
Married	57 (14.5)	11.00 (4.00)		
*Gender*				
Male	105 (26.6)	9.00 (4.00)	12,906.000	0.022*
Female	289 (73.4)	10.00 (4.00)		
*Parity*				
Yes	53 (13.5)	11.00 (4.00)	7441.000	0.037*
No	341 (86.5)	10.00 (4.00)		
*Educational level*				
Early (none, primary and secondary)	43 (10.9)	9.00 (5.00)	6295.000	0.074
Advanced (tertiary)	351 (89.1)	10.00 (4.00)		
*Age*				
Young adults (18–39)	358 (90.9)	10.00 (4.00)	5336.000	0.087
Older adults (> 40)	36 (9.1)	10.50 (3.00)		
*Ethnicity*				
Malay	296 (75.1)	10.00 (4.00)	12,827.500	0.084
Non-Malay (Chinese, Indian, Others)	98 (24.9)	9.50 (5.00)		
*Exposure to PPD*				
Yes	102 (25.9)	10.00 (5.00)	13,891.000	0.309
No	292 (74.1)	10.00 (4.00)		

*significant, p < 0.05

Moreover, knowledge scores among females were statistically significantly higher than males (*U* = 12,906, males = 105, females = 289, *p* = 0.022), with median scores of 10/20 (females) compared to 9/20 (males). Additionally, having at least one child was significantly associated with higher knowledge scores (*U* = 7,441, parity = 53, without parity = 341, *p* = 0.037) than having no children, with median scores of 11/20 (yes) and 10/20 (no).

#### Sociodemographic factors, postpartum depression awareness and attitudes

Sociodemographic variables and PPD exposure were associated with the attitude score for each group (Table 6). Median attitudes scores among males and females were 15 and 14 of 20, respectively; the distributions in the two groups differed significantly (Mann–Whitney *U* = 12,869, males = 105, females = 289; *p* = 0.021), which indicated a significant relationship between gender and attitude scores.

**Table 6:** Association between sociodemographic factors and exposure to PPD with attitude score using Mann Whitney U test (n = 394)

Variables	Freq., n (%)	Median (IQR)	Mann Whitney U	p-value
*Age group*				
Young adults (18–39)	358 (90.9)	14.00 (6.00)	5775.000	0.303
Older adults (> 40)	36 (9.1)	16.00 (8.00)		
*Gender*				
Male	105 (26.6)	15.00 (7.00)	12,869.000	0.021*
Female	289 (73.4)	14.00 (6.00)		
*Ethnicity*				
Malay	296 (75.1)	14.00 (6.00)	12,439.500	0.034*
Non-Malay (Chinese, Indian, Others)	98 (24.9)	15.50 (8.00)		
*Marital Status*				
Single	337 (85.5)	14.00 (6.00)	7560.500	0.010*
Married	57 (14.5)	16.00 (8.00)		
*Parity (Have at least a child?)*				
Yes	53 (13.5)	16.00 (8.00)	7298.000	0.024*
No	341 (86.5)	14.00 (6.00)		
*Educational Level*				
Early (None, primary & secondary level)	43 (10.9)	14.00 (6.00)	7276.000	0.701
Advanced (Tertiary level)	351 (89.1)	14.00 (7.00)		
*Exposure to PPD*				
Yes	102 (25.9)	16.00 (7.00)	12,219.500	0.007*
No	292 (74.1)	14.00 (6.00)		

*p < 0.05

Simultaneously, being a Malay (*U* = 12,439.500, *n*Malay = 296, *n*Non-Malay = 98, *p* = 0.034), being married (*U* = 7,560.500, *n*Married = 57, *n*Single = 337, *p* = 0.010), parity (*U* = 7,298.000, *n*Parity = 53, *n*No-parity = 341, *p* = 0.024) and exposure to PPD knowledge (*U* = 12,219.500, *n*Yes = 102, *n*No = 292, *p* = 0.007) with the attitude scores among social support networks during COVID-19 pandemic in Klang Valley.

#### Sociodemographic factors, postpartum depression awareness and beliefs

Table 7 depicted the relationship between sociodemographic variables and PPD exposure with PPD belief among social support networks during the COVID-19 pandemic in Klang Valley, Malaysia. None of the factors exhibited a significant connection with beliefs on PPD knowledge among social support networks.

**Table 7:** Association between sociodemographic factors and exposure to PPD with belief score using Mann Whitney U test (n = 394)

Variables	Freq., n (%)	Median (IQR)	Mann Whitney U	p-value
*Age group*				
Young adults (18–39)	358 (90.9)	12.00 (4.00)	6404.000	0.951
Older adults (> 40)	36 (9.1)	11.50 (4.00)		
*Gender*				
Male	105 (26.6)	12.00 (4.00)	15,067.500	0.916
Female	289 (73.4)	12.00 (4.00)		
*Ethnicity*				
Malay	296 (75.1)	12.00 (4.00)	12,826.000	0.084
Non-Malay (Chinese, Indian, Others)	98 (24.9)	11.00 (5.00)		
*Marital Status*				
Single	337 (85.5)	12.00 (4.00)	8803.500	0.310
Married	57 (14.5)	12.00 (3.00)		
*Parity (Have at least a child?)*				
Yes	53 (13.5)	12.00 (4.00)	8344.000	0.366
No	341 (86.5)	12.00 (4.00)		
*Educational Level*				
Early (none, primary and secondary level) Advanced (tertiary level)	43 (10.9) 351 (89.1)	11.00 (3.00) 12.00 (4.00)	6273.500	0.069
*Exposure to PPD*				
Yes	102 (25.9)	12.00 (5.00)	14,213.000	0.490
No	292 (74.1)	12.00 (4.00)		

*p < 0.05

### Effects of sociodemographic factors, knowledge, attitudes and beliefs on postpartum depression awareness

GLMM was run to predict PPD awareness levels among age, gender, ethnicity, marital status, parity, educational level, knowledge scores, attitude scores and belief scores. *p* values < 0.05 were considered statistically significant. Social support networks were allocated within the Klang Valley area and results revealed a statistically significant linear relationship between PPD awareness level and ethnicity which was the most potent predictor among the PPD awareness measures of respondents.

**Fig. 1:**
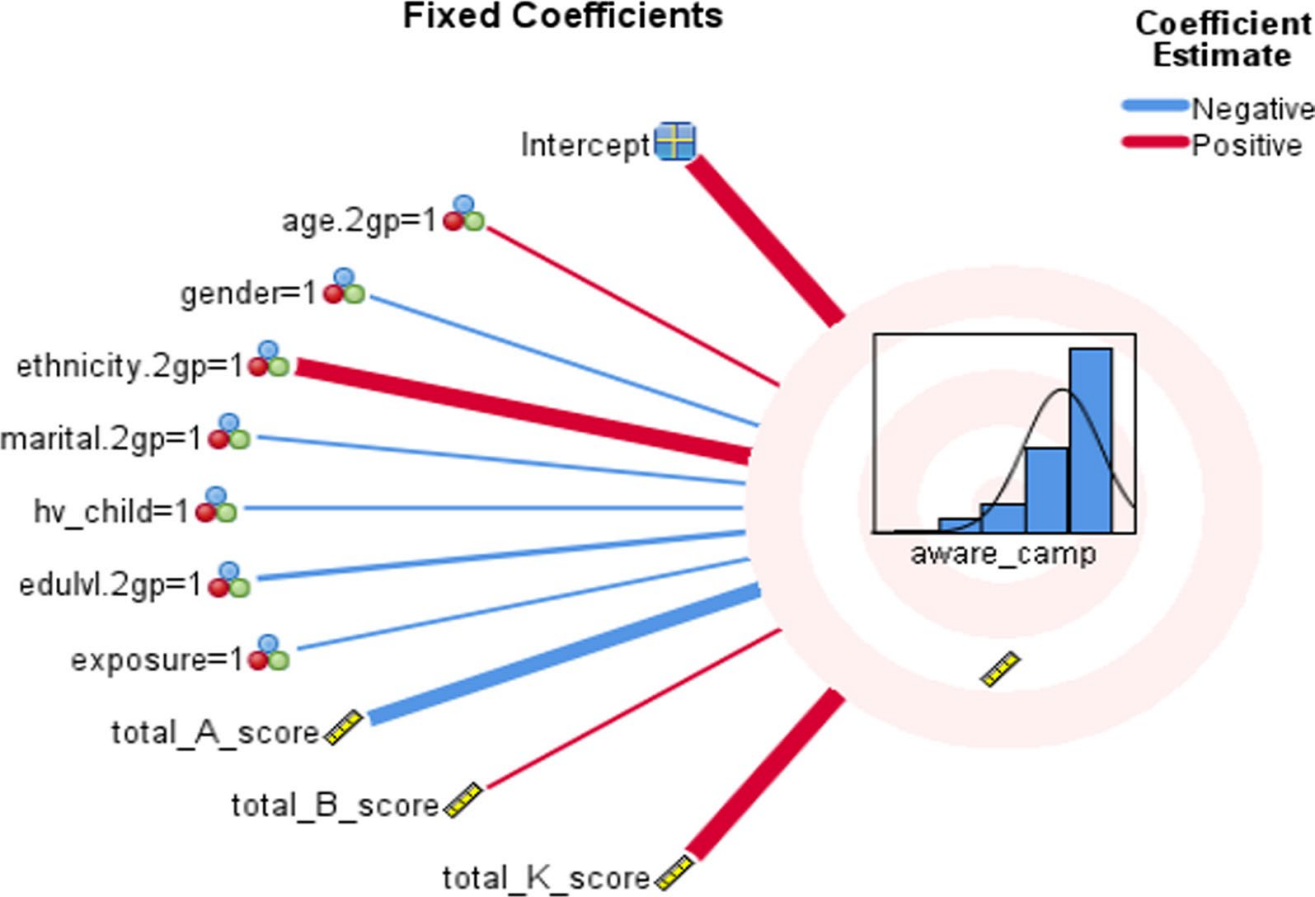
Thickness of the line of fixed coefficients showing the association between PPD awareness with sociodemographic factors (e.g. knowledge, attitude and belief scores)

According to analysis, being Malay (β1 = 0.403, 95% CI = 0.212–0.594, *p* < 0.001) is a significant positive predictor; the level of PPD awareness tend to increase by 0.403%. Additionally, total knowledge score (β1 = 0.077, 95% CI = 0.051–0.104, *p* < 0.001) is another significant positive predictor; for each increase in knowledge score, the PPD awareness level tends to increase by 0.077%. In contrast, total attitude score (β1 = − 0.020, 95% CI = − 0.032–0.104, *p* < 0.001) seemed to show as a significant negative predictor; for each increase in attitude score, the PPD awareness level will tend to decrease by 0.020% (Table 8, Fig. 1). Unexpectedly, no significant linear relationship was noted between PPD awareness level and total belief scores (β1 = 0.006, 95% CI = − 0.016–0.028, *p* = 0.605).

**Table 8:** Association between sociodemographic factors and exposure to PPD with knowledge, belief score using generalized linear mixed model test among social support networks in Klang Valley, Malaysia (n = 394)

Variables	Adj. coefficient	SE	t	Sig	95% CI	
					Lower	Upper
*Educational level*						
Early (none, primary and secondary)	− .184	.1327	1.386	.167	− .445	.077
Advanced (tertiary)	0*	−	−	−	−	−
*Exposure to PPD*						
Yes	− .083	.0949	− .872	.384	− .269	.104
No	0	−	−	−	−	−
*Age group*	.048	.2022	.238	.812	− .350	.446
Young adults (18–39)	0	−	−	−	−	−
Older adults (> 40)						
*Parity (Have at least a child?)*						
Yes	− .081	.3130	− .259	.796	− .697	.534
No	0	−	−	−	−	−
*Gender*						
Male	− .067	.0961	− .701	.484	− .256	.122
Female	0	−	−	−	−	−
*Ethnicity*						
Malay	.403	.0971	4.146	.000**	.212	.594
Non-malay (Chinese, Indian, Others)	0	−	−	−	−	−
*Marital status*						
Single	− .097	.2936	− .330	.741	− .674	.480
Married	0	−	−	−	−	−
Total belief score	.006	.0113	.517	.605	− .016	.028
Total knowledge score	.077	.0135	5.712	.000**	.051	.104
Total attitude score	− .020	.0061	3.221	.001**	− .032	− .008

Probability distribution: normal

Target: awareness

*This coefficient is set to zero because it is redundant

**Significant, p < 0.05

Only the main effects of these variables were tested because the current study is not interested in interaction effects. These results indicated differences concerning the knowledge level besides attitudes and beliefs adopted by social support networks during the lockdown based in a residential area in the Klang Valley. These differences were significant for Malay respondents in the case of sociodemographic factors. Additionally, respondents with more knowledge and fewer attitude scores tend to be more aware of PPD compared to others (Table 8).

The thickness of the line of fixed coefficients visualised the association between sociodemographic factors and PPD awareness with knowledge, attitude and belief scores. Only these variables were more explicit with significant positive effect (red line) or negative effect (blue line) on PPD awareness (Fig. 1).

## Discussion

This is a cross-sectional study that aimed to evaluate the social support networks' level of knowledge, attitudes and beliefs regarding PPD and examine their sociodemographic variables and exposure to the public information relating to PPD during the COVID-19 pandemic in Malaysia. The findings of this study showed that there was a significant positive knowledge, and a negative attitude and awareness level of PPD among social support networks for postnatal women.

Studies on the link between knowledge, attitude, and belief and PPD, particularly during Malaysia's Movement Control Order (MCO) period, are rare. According to previous investigations, there was a strong link between knowledge, attitude, and belief, all impacted by local sociodemographic characteristics like those found in Portuguese people [43] and Malaysian nurses [49].

### Sociodemographic characteristics of respondents

394 subjects took part in this research, which all were members of social support networks in the Klang Valley. The majority were women (73.4%), close to the general Portuguese population [43] who were shown to be more prevalent among women (88.1%). There is a chance that this was because the bulk of our responses were women, and many of the males thought our study was about PPD, a disease that only affects women. The vast majority of men who did not read the full description of our poll may have assumed that it was about PPD, and so skipped it. In addition, because the study's title referred to women, it drew in more female participants.

Those who answered the survey were between 18 and 60 years old, with the vast majority (90,9%) falling somewhere in the middle. These findings were also supported by the Portuguese research [43], which included young adults as participants (18–37 years old). This might be because our questionnaire was distributed mainly among young individuals rather than older persons on an internet platform. Because of this, young subjects were more easily reached via this platform than older people.

A further finding from this survey was that 75.1% of participants were Malay, compared to just 24.9% who were non-Malays. About 97% of the participants in prior research of nurses' knowledge, beliefs, and behaviors about PPD in Malaysia came up with the same result [49]. There is a chance this is because the Malay ethnic group makes up the vast majority of Malaysia's population. Most of the respondents were UPM students, the majority of whom were Malay, in addition to our research.

Additionally, most of those polled (85.5%) were single and childless (86.5%). Contrary to earlier research [49], which indicated that most respondents were married, another study found that the majority of respondents were single [43]. Our research varies from others previously stated since most of our respondents were students between the ages of 18 and 29, and the majority of these students were not married, as previously noted.

The same result can also be seen in the previous survey when 73.7% of the respondents had higher education, while most of our respondents had advanced educational levels (89%) [43]. Possibly because we delivered our questionnaires primarily to university students, most of whom had a tertiary degree. Another interesting finding from this study was that 74.1% of participants had never heard of PPD before participating in it, which might be explained by a shortage of public awareness campaigns or programs in the Klang Valley that specifically target it.

### Knowledge on post-partum depression

In our survey, most participants had a high degree of knowledge of PPD, with 76.4% scoring above the median. Our findings support this assertion. Some substantial gaps remained despite this. As a starting point, more than half of the respondents accurately identified the frequency of PPD during the COVID-19 pandemic, which contrasted with earlier research in different ethnic settings [43, 52]. This study's findings differ from previous ones since it was done during the COVID-19 epidemic. It seems from the results of our research that people in Malaysia and other countries were aware of the effects of the COVID-19 pandemic on mental health since Movement Control Orders (MCO) was used throughout the pandemic. This pandemic's travel and self-isolation limitations lead to a sedentary lifestyle and decreased mental health, both of which enhance the prevalence of PPD [42, 53].

First of all, most of our respondents answered questions on PPD signs and symptoms correctly, which indicates that they were aware of the warning signals of PPD, which is consistent with earlier research [43]. Most people thought that women with PPD only considered harming themselves. Self-harm is regarded as a warning sign of PPD. In contrast, women with PPD are more likely to suffer from poor moods, sleep disturbances, changes in food, diurnal variations in mood, loss of focus, and easily irritated [54, 55]. Postpartum women's support networks need to be well-versed about PPD symptoms so they can recognize the presence of likely symptoms early on, helping them to diagnose PPD earlier and urging them to seek professional treatment [43].

PPD is associated with a higher chance of developing in women who have a history of psychiatric disorders or do not desire to get pregnant [43]. There was still a lack of knowledge regarding PPD's causes among our respondents. Postpartum women can acquire the disorder even if they have no prior history of mental health issues [54]. Like earlier research [43], most of our respondents were able to identify effective treatments for PPD. However, more than half of those polled thought that a general practitioner could not detect PPD in women. As a result, many of our survey participants had no idea how important it is for doctors to catch PPD in its early stages.

### Attitude on post-partum depression

During the COVID-19 pandemic in Klang Valley, the attitude of social support networks toward PPD was examined primarily by descriptive analysis. Results indicated that most respondents had a favorable attitude toward PPD, with only 36.2% scoring over the median score for a positive attitude toward PPD. Additionally, a poll among Portuguese citizens found that a sizable percentage of respondents had a favorable opinion of PPD [43].

According to our findings, 44.4% of participants and 25.0% of participants in a prior study among the Portuguese population believed that PPD was frequent [43]. A frequent misconception about baby blues is that it occurs in postpartum women owing to hormonal imbalances [14, 43]. This attitude may reflect this. When a woman and her social support network are unable to identify PPD symptoms, it makes it difficult for physicians to assist treat this disease in an effective manner [43, 47], as a result, it is imperative that this attitude be clarified. After that, most participants believed that women are born with how to care for a newborn properly.

Around one-fourth of the sample previously believed that women intuitively had the abilities and knowledge required to care for a baby in this study, making it more difficult for postpartum women to recognize signs of PPD. Postpartum women may downplay the severity of their PPD symptoms, making it more difficult for them to get the treatment they need to manage their condition [43, 45].

Most participants believe that women with PPD do not choose to have PPD and that all new mothers should be screened for the condition. The response indicates respondents' positive attitude about the emotional assistance women require from their social support networks when they have PPD and the necessity of early PPD screening for postpartum women suffering.

### Belief on post-partum depression

The vast majority of our survey participants had a favourable belief on PPD (84.8%), with a vast gulf between those who have a favourable idea and those who hold a negative thought. An Australian study found that 73% of respondents had a positive belief in PPD, showing that most Australians did not underestimate the significance and occurrence of this mental health problem in postpartum women [45].

About 47.2% of those polled had a neutral opinion on whether PPD women should be encouraged to seek alternative therapy. Most respondents in previous research [45] favoured non-pharmacological interventions, including counselling and talk therapy over antidepressant medication, and more than half of respondents felt that PPD required specialized treatment. A study by Sealy et al. [47] found that only 10% of respondents said they received encouragement from non-professional sources such as family/friends and church/support groups, but most respondents (85.2%) said they trusted their physician/obstetrician for information and avoided misconceptions about PPD, especially among the mother's social support network [47].

On average, 48.7% of those polled (192 out of 394) also said it was challenging to receive PPD therapy during the COVID-19 epidemic. Beliefs like these might impact women's prenatal and postnatal care and satisfaction levels when they need complete family support to perform an intervention [26]. Despite the high incidence of PPD, the majority of those affected did not seek professional help [55]; multiple factors may have contributed, but the most likely one conflicted with the partner during the postnatal period or, in other words, a low level of support [26]. Studies have shown that social support networks including spouses, family and friends who know and believe in the presence of PPD might motivate women to seek assistance and treatment for professional care, which was also backed by additional research [47].

According to the findings, PPD is widely believed to be a hoax and not fatal by most respondents. Additionally, it was found in research that PPD can significantly impact both the woman and her infant's growth and development [56]. After that, the study found that only 51.9% of respondents believed in PPD and were aware of its severe symptoms, including sorrow. This is likely owing to respondents' demographic profiles and opinions of PPD, which are mixed [47]. More than half of respondents in Australian research disagreed with the claim that PPD is not a severe mental health condition (59%) [45].

### Association between sociodemographic factors with knowledge

According to our findings, awareness of PPD was significantly correlated with gender, marital status, and parity. In comparison to male respondents, female respondents knew more about PPD. In a study of the general Portuguese population, it was found that women had more awareness about PPD, which was comparable to this conclusion. Because PPD is more closely associated with females, there may be variations in how men and women see it. Thus, women may feel compelled to learn about PPD features to recognize and diagnose it correctly [43].

As a result, married individuals had better awareness of PPD than those who were single, and this connection was statistically significant. In earlier research, married or common-law people had more understanding of postnatal mental health than single, widowed, or divorced people. This conclusion echoed other findings [46]. PPD, according to the World Health Organization, is a mental illness associated with pregnancy and the six weeks following childbirth [1, 16]. Because PPD occurs after pregnancy, married people are more familiar with PPD because it is a mental illness that affects moms [6].

There was a statistically significant link between awareness of PPD and parity in Branquinho study [43], as individuals who have children are more knowledgeable than those who do not have children. Research shows that those who have young children at home have a better understanding of PPD. Concerning PPD, new mothers may engage in more informing behaviors (e.g., searching online for information on PPD, consulting with health experts) because of the perceived immediate significance of the condition.

There was no correlation between age and PPD knowledge in the study. According to Branquinho [43], older people had lower knowledge about PPD than younger people because older people were more likely to have less schooling. Our findings differ from those of the previous study, which might be explained by the fact that most older individuals who participated in our research were female. For this reason, women are more likely than males to have a solid understanding of PPD, which has an impact on our findings.

Results of GLMM analysis showed that educational level was not a significant predictor of a good understanding of PPD, in contrast to Branquinho’s study who reported that the more educated people had better knowledge of PPD than less educated people [43]. However, less educated people may have had fewer opportunities to learn about mental health problems in general, particularly PPD, which could lead to a lack of knowledge about PPD.

### Association between sociodemographic factors with attitude

Six key sociodemographic variables impacted the attitudes of social support networks toward PPD. They were the following: marital status, gender, number of children, age, ethnicity, and education levels are all considered. A Generalized Linear Mixed Model was used to modify the findings of the connection between sociodemographic variables and social support networks' attitudes about PPD. There was a correlation between the attitude score among social support networks during the COVID-19 epidemic in Klang Valley and the demographics. These findings were backed up by previous research that found a link between views regarding PPD and factors like gender, marital status, and parity.

Then, our results also showed no significant association between age group and educational level with a total attitude score. These results contrasted with a prior study, which revealed a strong link between educational attainment and attitude regardless of age [43]. Ethnicity, gender, and parity were significant predictors of views about PPD in our research. Age and educational level were previously found to be determinants of attitudes about PPD in GLMM. This new finding contradicts the finding of Branquinho’s study.

According to the research of the Portuguese population, males are more likely than women to have a negative attitude toward premenstrual dysphoria (a higher attitude score). According to these studies, women may have a more challenging time recovering from PPD since they require moral support and sound counsel from their male partners to persuade them to seek professional assistance [43, 45]. To avoid revealing their emotional issues with anybody around them, women may begin to believe that their social support network has a negative attitude about PPD, which may prevent them from seeking treatment in the future [43].

The Mann Whitney U test and GLMM in our study indicated no significant relationship between educational level and attitude toward PPD. This finding contrasts with that of research conducted among the Portuguese population, which found that those with lower levels of education had a more unfavorable attitude about PPD [43, 45]. In terms of parity, we found that parents with children have a more positive attitude regarding PPD than parents without children. Earlier research shows that parents have a more negative attitude than non-parents [43]. Using the Mann Whitney U test, it was discovered that married respondents have a more positive attitude regarding PPD than unmarried respondents. According to Branquinho, married or cohabiting people had a more pessimistic view of PPD [43]. Our study found that non-Malays have a more pessimistic view of PPD than Malays in terms of ethnicity.

### Association between sociodemographic factors with belief

Results showed no correlation between sociodemographic factors (age, gender, ethnicity, education, parity, and marital status) and the level of belief in PPD among social support networks during the COVID-19 pandemic in Malaysia's Klang Valley. This is because of the lack of statistical significance between the two variables. A study in the Mexican population found a link between Ethnicity identification and PPD belief in providing social assistance to improve quality of life, such as customary celebrations, christenings, and social activities [26]. The discrepancy in findings was most likely caused by the study's small sample size of various ethnicities and backgrounds.

In Australian research, no significant variations were found between the sexes regarding beliefs about PPD's symptoms, such as exhaustion, or its causes, such as not being able to cope with the baby's needs and feeling stressed and under pressure, especially [45]. Having a negative belief about PPD was related to being male in another study [55], which may be because men are less exposed to, knowledgeable about, and aware of PPD [57].

Next, when questioned about the most severe mental health issues during pregnancy, there was a strong correlation between the Australian population's age group and belief in PPD [45]. In another study, researchers found a link between respondents' age and their beliefs on PPD, finding that the younger generation had a more pessimistic view of the condition, most likely because they had not heard of it before becoming postnatal mothers [55].

According to the results of one study, having children was linked to a more favorable perception of PPD because of the primary instruction they've gotten on the subject. The study's selection impact, which focused more on the influence of belief on PPD in the family structure that having children in their family's environment has an inconsistent connection with the results, was observed [47]. When it came to the belief in PPD, there was no correlation between literacy score and primiparity or multiparity, according to one study [58].

The notion that only PPD necessitates medical attention was unaffected by educational level in a recent study [47]. According to the results of another study conducted among pregnant and postnatal women, there is a strong correlation between educational attainment and PPD literacy, such as the view that increased exposure and raising awareness via a community campaign are to blame [58].

### Exposure to PPD with the knowledge, attitude and belief

In the first place, we found that among social support networks during the COVID-19 pandemic, exposure to PPD was a strong predictor of knowledge of PPD. Other research has shown that exposure to PPD leads to greater awareness about PPD. It has been shown that nurses who have had less experience with PPD therapy are less knowledgeable about the condition's treatment and symptoms, according to a study performed among nurses [49].

A substantial correlation was found in social support networks between PPD exposure and attitude toward PPD attitudes based on the Mann Whitney U test and GLMM. Research done among Portuguese people found no link between exposure to PPD and attitudes about PPD; however, this finding contradicts that [43]. GLMM results also showed that participants who were not exposed to PPD had a lower attitude score towards PPD compared to participants who were exposed to PPD, but this result is not supported by any previously conducted surveys. In our study, exposure to PPD also acts as a predictor variable of attitudes towards PPD.

A large majority of respondents in our study said they had no prior exposure to PPD, which is consistent with a survey of Portuguese citizens, even though most people viewed these campaigns as either helpful or extremely helpful [43]. Not only that, but prior research has shown that being exposed to PPD resulted in a more positive view toward the substance in question [43, 46]. Consequently, these outcomes indicate that public education efforts regarding PPD should be funded to increase awareness of the disease.

Beliefs of PPD were not affected by exposure to public information since there was no statistically significant difference, while there was one non-exposed group of respondents that exhibited substantially different literacy scores, such as belief in PPD, based on Mirsalimi’s research (N = 692) that specified the variable "Source of seeking information on PPD" [58]. Moreover, according to a study conducted in Pakistan, postnatal mothers who are illiterate were more likely to believe in PPD [59]. According to the results of Australian research, PPD was more likely to be recognized and accepted as a mental health issue in pregnant women with a professional mental health background [45]. Further research also found a link between PPD exposure and the public's perception of PPD [55]. Our findings in this study differed from other studies probably due to numerous reasons: a) prolonged restriction of movement during pandemic periods in Klang Valley that would limit the exposure towards mental health problems; b) Random Error, which the "actual" value of a variable varies randomly throughout a study population that has the potential to mislead measurement results and skewed the true relationship in favour of the null [60]; and c) comparing the P values of two studies with identical sample sizes and no errors in measuring two separate relationships shows the association with a bigger magnitude of effect (e.g., difference between groups) and will have a lower P value [61].

## Conclusion

We may conclude that during the COVID-19 pandemic in the Klang Valley, most individuals demonstrated good understanding, with negative attitudes about PPD. Marital status, gender, and parity all had a significant impact on one's understanding of PPD. Ethnicity, gender, parity, and education levels were all shown to be significantly associated with one's attitude toward those with developmental disabilities.

The sociodemographic variables did not affect the participants' PPD beliefs, as well as public awareness of PPD that, in contrast, had a significant impact on participants' attitudes and knowledge about the disease. PPD awareness is poor among Malaysian adults; nevertheless, there is a significant interest in learning more about PPD through public education initiatives. It follows that these findings might serve as a starting point for further research into how to better educate the public about PPD during COVID-19 pandemic.

Women who have just given birth are more likely to suffer from PPD than others, and those mothers suffering of depression require emotional assistance. It might be helpful for women's social network to recognize that PPD is common, and that many other women have felt the same way. Healthcare providers must try to get in touch with the woman's relatives and explain to them why they need to step in and help out, and should make sure that she and the baby are receiving the attention they need.

When it comes to women's health, some family and even some health care providers may not take their worries seriously, which develop feelings of inadequacy for postnatal women. In certain cases, postnatal women may not be able to adequately care for both themselves and their babies, beside to their own specific needs, and this is true especially for teenagers.

Spouses and family members should pay attention to the postnatal woman and be understanding of her situation, and they should be encouraged to provide postnatal women practical and emotional help and reassuring words. A woman who is having difficulty caring for her baby should never be abused in any way, whether it verbally or physically.

To ensure that women who are suffering of depression and their families are receiving the help they need, it is important to have frequent follow-ups with them and their families. The level of support that women anticipate from their social networks might be influenced by their own expectations of that assistance. The findings of this research recommend that assessing social support network of postnatal women needs and expectations is critical to their recovery after delivery. A mother's postpartum recovery might be aided by treatments that increase the capacity of mothers to mobilize their social networks.

### Implication for practice

We found that respondents had a low degree of understanding of postpartum depression, particularly among males. It is thus advised that the Ministry of Health (MOH) or any other Non-Governmental Organizations (NGO) hold awareness campaigns and public education on this mental health problem among postnatal moms. Postpartum care should include mental health screenings and counselling sessions for the new mother in order to guarantee the healthy development of both the baby and their mothers.

As a result of our research, we would want to encourage other researchers to perform a similar study to establish the degree of knowledge, attitudes, and beliefs about postpartum depression among social support networks in various Malaysian areas or states. A non-pandemic scenario might also be used to study the effects of the lockdown on knowledge, attitude, and belief about postpartum depression. As well as, the study's findings may be expanded by other researchers who can examine the knowledge, attitude, and belief of women about postpartum depression based on other characteristics such as income or profession (healthcare or non-healthcare workers).

It's also important that social support networks be well-informed on PPD, its impacts, and the best ways to treat it. Campaigns to raise awareness of postpartum depression and its risk factors, as well as to promote a healthy lifestyle and offer adequate environmental support for postnatal mothers, should be conducted more effectively in society. A wonderful strategy to attract attention to this mental health concern among postnatal women is via educational displays and activities. Additionally, it may be a powerful tool in educating women about the dangers of PPD, including the origins, symptoms, risk factors, and treatment options.

### Strengths and limitations

This is one of the early studies on the area of PPD in Malaysia region during COVID-19. This cross-sectional research was able to determine the relationship between sociodemographic variables and exposure to public information and the degree of knowledge, attitude, and belief about PPD. A lot of data has been collected using low-cost approaches thanks to the use of self-administered surveys through Google Forms in this research.

There were several limitations to this study. To begin, we limited our sample attributes of social support network in the Klang Valley and its surrounding areas, so findings may not be generalized to the entire Malaysian population. Secondly, data collection was conducted at the time of movement control order (MCO), as the survey was circulated via WhatsApp, Facebook, and other social media sites, so it was pretty likely that most people, especially the elderly, would disregard it, and younger respondents with higher educational levels responded more, which leads to non-response bias. It is also possible that respondents misunderstood the questions because we could not clarify them face-to-face. Finally, because our study was cross-sectional, we were unable to draw causal or temporal conclusions in the absence of repeated measures.

## Data Availability

The datasets used and analyzed during the current study are available from the corresponding author on reasonable request.
